# A recent regulatory storm in China: theoretical interpretation and applicability

**DOI:** 10.1007/s44216-023-00011-3

**Published:** 2023-03-20

**Authors:** Youyi Zhang, Sen Gong

**Affiliations:** 1 Development Research Center of the State Council, Beijing, China; 2 Center for International Studies on Development and Governance at Zhejiang & Zhejiang University, Hangzhou, China

**Keywords:** Regulatory storm, People-centered approach, Stakeholder economy

## Abstract

While the recent “regulatory storm” in China has attracted attention from media, academia, and policy makers both in China and overseas, there lacks theoretical interpretation of Chinese central government’s major regulatory practices. Based on the review on global reflection of the shortcomings of the current market economy system and China’s own market economy practices, this article argues that China’s strengthening of market regulation is based on a people-centered paradigm of the state-market relationship that takes into account both efficient market and effective government and that is consistent with the international framework to improve the market economy system, in particular the stakeholder economy. To support the argument, this article illustrates China’s “regulatory storm” in terms of corporate governance, environmental goals, social impacts, adverse consequences, and employee rights with reference to cases from sectors affected by the “regulatory storm”. Moreover, China’s recent regulatory measures reflect the effectiveness of the “strong government” in rapidly rectifying market order and are in line with the global trend of building a new market system, which is worthy of reference for other Asian countries with similar political and economic contexts.

Since the end of 2020, China’s central government has adopted major regulatory measures pertaining to a number of industries, often referred to as a “regulatory storm” by the outside world. The “regulatory storm” has positive achievements, but there have also been some misunderstandings in China and abroad, and these measures have even been misinterpreted by some foreign media as crackdown on private enterprises and as “state capitalism.”

Currently, most of the interpretations of and studies on the “regulatory storm” address specific issues and industries, lacking a comprehensive perspective. From the perspective of the state-market relationship, this article argues that China’s strengthening of market regulation is based on a people-centered paradigm of the state-market relationship that takes into account both efficient markets and effective government and that is consistent with the global efforts to overcome the shortcomings of the current market economy system. The regulatory storm could have some implications for other Asian countries with similar political and economic systems.

## Research background

Since the listing of Ant Financial was suspended in November 2020, the Chinese government has implemented major regulatory measures in a number of industries. Over time, relevant line ministries of the Chinese government have successively introduced regulatory measures in various areas, including the platform economy, educational training, and information security. These major regulatory measures have achieved some positive results, but there are also some misunderstandings and doubts. For example, some Western media and experts believe that the Chinese government is suppressing private capital and “local tyrants” and engaging in “state capitalism.” The Economist, for example, published an article in July 2021 that maintains that China’s regulatory measures show a shift toward curbing private capital. Dalio, the founder of Bridgewater Associates, argues that China is embarking on “true state capitalism” by shifting to support the development of key technologies and strengthening the hard power of its economy to catch up with the US and the West. The Financial Times published an article in August 2021 that claims that China’s “crackdown” on the private economy does not respect the laws of the market and poses significant risks to foreign investors investing in “China concept stocks”. Reuters suggests that one of the largest impacts of recent regulatory measures is the uncertainty they create, especially for foreign enterprises; these institutional changes are part of a larger paradigm shift in China’s ambition to become technologically self-sufficient and a technological superpower. Some groups in the Philippines and South Korea suggest that China’s measures have had a negative impact on employment and development in some industries such as entertainment and English language training. Foreign media attention to China’s “regulatory storm” has remained high; for example, the Wall Street Journal has been running a column and following up stories since December 2020, with mostly negative coverage.

China’s official explanation is that some market activities do not conform to the “new development philosophy” proposed by President Xi Jinping. The People’s Daily commented on September 8, 2021, that “this series of regulatory measures is a pragmatic action to promote the formation of a fair and competitive market environment and better protect consumers’ rights and interests using a strategy that involves regulating market order, building a new development dynamic, and promoting high-quality development and is a powerful initiative to strike a balance between development and security, between efficiency and fairness, between vitality and order, and between domestic and international affairs as well as to improve the socialist market economy system in a complex and changing situation.”^1^ In response to the abovementioned misunderstanding by the international community regarding major regulatory measures, the relevant ministries of the Chinese government urgently addressed the issue, but it has not yet eliminated misunderstanding and uncertainty. Given low degree of mutual trust between China and the West in recent years, the theoretical foundation and policy implications behind China’s “regulatory storm” need to be further clarified. From the perspective of the theoretical foundation, it is necessary to clarify that China’s strengthening of market regulation is based on people-centered philosophy of the state-market relationship and is also in line with the global efforts to overcome the shortcomings of the current market economic system, including in Western countries, and has some implications for other Asian countries with similar political and economic systems. In terms of policy implications, this article suggests that it is necessary to take into account the perspectives of both new global efforts and China’s new concept for development, to interpret the new measures of Chinese market regulation in a common international discourse and through Chinese policies, and clarify the direction and boundary of government regulation to the international community, so as to eliminate the above-mentioned misinterpretations and boost the confidence of domestic and foreign investors.

## Literature review

### Global reflection of the shortcomings of the current market economy system

In recent years, Western countries have experienced a collective trend of new interventionism and a readjustment of state-market relations. Influenced by the global financial crisis, Brexit, and Trump’s rise to power, there is a reflection on the lack of development of neoliberalism, a rising call for more state intervention in the economy, and an increase in the number and intensity of state intervention measures within Western countries. Since the pandemic, economic and social inequalities have become prominent, further driving the rise of new interventionism. Internationally, new reflections of the shortcomings of the current market economy include both amendments to the existing institutions and the introduction of new concepts. The adjustment of existing market institutions consists of industrial policy, antitrust and government regulation (which is the focus of this article), and corporate taxation, and the introduction of new concepts is mainly reflected in the updating of the concept of the “stakeholder” market economy by Schwab (2021), the founder of the World Economic Forum.

#### Some amendments to the existing market institutions

Since the 1980s, under the neoliberal paradigm, new theoretical exploration and practical innovations such as responsive regulation, smart regulation, and regulatory cooperation have emerged in Western countries, but none of them have broken through the existing market-centered and market-led paradigm.

##### Responsive regulation theory

Ayres and Braithwaite (1992) put forward the theory of responsive regulation, which proposes the combination of governmental and nongovernmental means to carry out market regulation. Yang (2014) argues that the core of responsive regulation theory includes focusing on the regulation of key issues that need to be addressed, prioritizing soft measures such as persuasion, education and self-regulation, using the strongest regulatory measures as the last resort, and gradually increasing the intensity of regulation in line with real needs. This theory focuses on how regulators can effectively regulate through effective responses, with the intensity of regulation ranging from “soft” to “hard”.

##### Smart regulation theory

Gunningham (1998) advocates the implementation of diversified forms of regulation on the basis of government regulation, the adoption of flexible and innovative forms of social regulation such as self-regulation and co-regulation, and the use of commercial institutions and nongovernmental organizations as regulatory agents, while improving the effectiveness and efficiency of more traditional forms of direct government regulation. This theory argues that the use of multiple rather than single policy instruments and the inclusion of a wider range of regulatory actors will enhance the quality of regulation. The focus of this theory is to develop a combination of regulatory tools and regulatory participants that are appropriate to the specific context.^2^

##### Agile governance theory

According to the World Economic Forum (2018), agile governance aims to shift the manner in which policies are generated, deliberated, enacted and enforced in the Fourth Industrial Revolution. Agile governance is defined as adaptive, human-centred, inclusive and sustainable policymaking, which acknowledges that policy development is no longer limited to governments but rather is an increasingly multi-stakeholder effort.

##### Regulatory sandbox theory

In response to new issues facing the regulation of emerging areas such as fintech, the UK Financial Conduct Authority first introduced the concept of a regulatory sandbox in 2015. The Authority defines the regulatory sandbox as a safe space that is determined by the regulator and where enterprises can test innovative products and services, new business models, and payment mechanisms, and even if the relevant enterprises, products, and behaviors violate existing laws and regulations, they will not immediately suffer the corresponding regulatory consequences. The regulatory sandbox provides innovators with the opportunity to gain regulatory expertise.

The above regulatory adjustments are efforts to address the weakness of current market institutions in Western countries, but in general, they are minor fixes to the market-centered neoliberal model.

#### Some revolutionary concepts

The revolutionary concepts are mainly reflected in the updating and practice of the concept of “stakeholder economy”. According to Schwab, there are currently two market economy systems prevailing in the world: one is the shareholder-led market economy in the US and the West, and the other is the government-led market economy in many emerging economies. Both systems have generated tremendous economic progress, but both have also had negative social, economic and environmental impacts, leading to increased inequality in income, wealth and opportunity as well as greater tensions between high- and low-income earners. In a new book published in 2021, Schwab (2021) advocates and updates his “stakeholder” market economy concept developed in the 1970s, arguing that the core of this concept is that enterprises not only focus on maximizing the short-term profits of investors but also seek long-term value, taking into account the needs of both all stakeholders and society. With the globalization of the economy, the market economy has morphed into one that focuses only on the profits of investors; in the face of today’s social, economic, health and environmental crises, the best way forward is to promote the “stakeholder” market economy system and place the interests of all people and the world at its center.

The concept of the “stakeholder” market economy essentially breaks away from the market-centered neoliberal model by no longer focusing on the maximization of short-term profits of investors and aiming to achieve balanced economic, social, and environmental development. McKinsey & Company has borrowed this concept and proposed a framework of principles that enterprises should follow in terms of corporate governance, environmental goals, social impacts, adverse consequences, and employee rights (Table 1) that is highly recognized and accepted by the international business community.

**Table 1:** McKinsey’s framework of stakeholder principles

Basic elements	Corporate Governance	Environmental goals	Social impacts	Adverse consequences	Employee rights
**Specific connotation**	Focus on the interests of multiple parties, add corresponding independent directors, and prioritize the interests of stakeholders rather than investors	Formulate and implement environmental protection goals	Enhance suppliers’ capacity to promote their care for economic, environmental, and social impacts; promote inclusive development and strengthen interaction with communities	Serve the long-term needs of consumers and eliminate the foreseeable negative consequences of products	Respect employees’ rights and invest in their future

Source: The official website of McKinsey & Company

### China’s adherence to a new form of state-market relationship that insists on the supremacy of the people and considers both an “efficient market” and “effective government” since the 18th National Congress of the Communist Party of China (CPC)

#### Discourse of the CPC and the government on the new state-market relationship

The most essential feature of the Chinese state-market relationship in the new era is to insist on the supremacy of the people, to insist that the purpose of the socialist market economy is to enhance the overall well-being of the people, and to insist that the government is active, responsible, and fair in its governance without being captured by any capital and interest groups. Adhering to the people-centered philosophy of development is an important part of Xi Jinping Thought on Socialism with Chinese Characteristics for a New Era. Xi first put forward the people-centered philosophy of development at the National Conference on Propaganda and Ideological Work in August 2013. In October 2015, Xi proposed, at the Fifth Plenary Session of the 18th CPC Central Committee, that “we must adhere to a people-centered philosophy of development and take the promotion of people’s well-being and all-round development as the starting and ending points of development.” In October 2017, Xi pointed out in the report of the 19th National Congress of the CPC that the socialism with Chinese characteristics for a new era must adhere to the people-centered philosophy of development. In July 2021, Xi noted in his speech at the meeting celebrating the 100th anniversary of the founding of the CPC that “the Party has always represented the fundamental interests of all Chinese people; it stands with them through thick and thin and shares a common fate with them. The Party has no special interests of its own—it has never represented any individual interest group, power group, or privileged stratum”.

The new development philosophy is people-centered. In October 2015, the Fifth Plenary Session of the 18th CPC Central Committee put forward the new development philosophy of “innovative, coordinated, green, open, and shared development.” China’s economy has shifted from the high-speed growth to high-quality development. High-quality development reflects the new development philosophy, development in which innovation is the driving force, coordination becomes the endogenous feature, greenness becomes the universal form, openness becomes the necessary path, and sharing becomes the fundamental purpose, the essence of which is to achieve comprehensive and coordinated economic, social, and environmental development.

Under the guidance of the people-centered philosophy of development and the new development philosophy, the CPC and the government have proposed a paradigm of a state-market relationship with Chinese characteristics that considers both an “efficient market” and “effective government.” In November 2015, Xi stated in his speech at the 28th collective study session of the Politburo of the 18th Central Committee with an emphasis on having both an “efficient market” and an “effective government”.^3^

Under the paradigm of a state-market relationship with Chinese characteristics that takes into account both an “efficient market” and “effective government,” the CPC and the Chinese government have further clarified market regulation measures. In December 2020, the Central Economic Work Conference clarified that antitrust and anti-unfair competition are inherent requirements for improving the socialist market economy system and promoting high-quality development. In March 2021, the ninth meeting of the Central Finance and Economics Committee stated that it is necessary to clarify rules, draw bottom lines, strengthen regulation, regulate order, better coordinate development, security, and domestic and international affairs, promote fair competition, oppose monopolies, and prevent the disorderly expansion of capital. The 23rd meeting of the Central Committee for Comprehensively Deepening Reform in December 2021 emphasized the need to strengthen antitrust and anti-unfair competition, aiming to remove market barriers, and improve the efficiency and fairness of resource allocation; meanwhile it emphasized to accelerate the transformation of government functions, aiming to improve the effectiveness of government regulation, and promote a better combination of an efficient market and effective government. The Central Economic Work Conference in December 2021 put great emphasis on the need to give full play to the positive role of capital as a factor of production while effectively controlling its negative role, and that it is necessary to set up “traffic lights” for capital, to strengthen the effective regulation of capital in accordance with the law, and prevent the unrestrained growth of capital. In February 2022, Xi, chairing the 24th meeting of the Central Committee for Comprehensively Deepening Reform, states that it is necessary to promote a better combination of effective government and efficient market, improve government supervision and service efficiency, protect and stimulate the vitality of enterprises, pay attention to maintaining a fair market environment for competition, and promote more outstanding enterprises to stand out in market competition.

#### Discussions by Chinese academia on the new state-market relationship that considers both an “efficient market” and “effective government”

Chinese academia argue that “effective government” provides a good basis for implementing “strong regulation.” Lin and Liu (2021) maintain that the role of an efficient market and effective government as “two hands” should be addressed, especially a focus on the government playing the role better, and that the government should manage the macro-economy effectively from the practical point of view.^4^ According to Zhang (2021), based on the dialectical relationship that an effective government is based on an efficient market, an efficient market is led by an effective government, it is necessary to strike a balance between an efficient market and effective government through the Party’s overall leadership of the development of the socialist market economy system with Chinese characteristics and the guidance of the new development philosophy. Chang (2021) argues that the real essence of building a high-level socialist market economy system lies in promoting the market’s efficiency with the government’s effectiveness and, by fulfilling its due responsibilities, the government promotes the market to serve its role correctly, that is, “the power that should be delegated to the market and society must be released to the full and in the right place, and the government must be in charge of things that it must manage to its full capacity.” Liu and Zang (2021) note that the main goal of regulation is to facilitate fair competition in the market and that it is necessary to promote the fair participation of different market players in market competition by strengthening antitrust and anti-unfair competition enforcement and by effectively carrying out fair competition reviews.

Some scholars maintain that the “strong regulation” policy should be improved. According to Huang et al. (2022), the purpose of “strong regulation” is to retain benefits and solve problems. However, if a policy simply follows traditional economic thinking, it is likely to result in “throwing the baby out with the bath water,” which is not conducive to the balanced development of the platform economy.

In general, there is still a lack of in-depth discussion of a regulatory model with Chinese characteristics that takes into account the common international features, which is adapted to the changing contexts including the new requirements of the new development philosophy and the new business forms, such as the platform economy. Wang (2021) argues that there are “three more and three less” problems in the Chinese research on regulatory theories, i.e., more studies on foreign regulatory theories but less on regulatory issues in the context of Chinese institutional environment, more on regulatory laws, regulatory agencies, and regulatory performance in a scattered manner but less on the regulatory system as a whole, and more on traditional regulatory policies but less on incentive regulatory policies. The existing regulatory theories in China are still not well adapted to the needs of regulatory practices of the Chinese government in the new era.

## A new theoretical framework

This article argues that the major regulatory measures of the Chinese government are a pioneer in the global reflection of a new market system in the new era and are highly compatible with the international framework. China’s “regulatory storm” not only is in line with the new global exploration of improving the market economic system, especially the international framework of the “stakeholder” market economy, but also is based on the paradigm of a state-market relationship with Chinese characteristics that takes into account both an “efficient market” and “effective government” and is a new approach to the new market system.

### Main features of China’s regulatory storm

Compared with slight adjustments to the existing market economy model in Western countries, the current “regulatory storm” in China has the following features:

- First, it adheres to a people-centered approach and focuses on public interests. In this “regulatory storm,” China insists on the supremacy of the people, improves the overall well-being of the people, comprehensively strengthens environmental, social, and governance (ESG) regulation, ensures a balance between the interests of the public and investors, and places regulatory policies under the framework of common prosperity that reflects the essential requirements of socialism and the important characteristic of Chinese-style modernization.
- Second, it covers a wide range of areas and industries. Traditional regulation theories divide regulation into three categories: economic regulation, social regulation, and antitrust regulation. This “regulatory storm” is not limited to a specific area but to a combination of the above three means. It covers various aspects and industries of the economy, society, and environment. Since the end of 2020, the key areas of regulation have included Internet finance, educational training, online games, entertainment, real estate, and banking, among others, with a significant and far-reaching impact.
- Third, the intervention is strong and effective. In this “regulatory storm,” the Chinese government has resolutely taken necessary regulatory measures, especially quick-acting administrative measures, including “scorched earth” tactics, a term commonly used by foreign media, and achieved some quick results. In terms of educational training, the number of New Oriental schools decreased from more than 1600 to about 800 in fiscal year 2021. In terms of technology giants, Tencent’s revenue growth fell to the lowest level in nearly 20 years according to the Wall Street Journal (Zhai and Wang 2022).^5^
- Fourth, the transition period is short. Once a regulatory loophole is discovered, it is corrected immediately and resolutely. Taking the platform economy as an example, regulatory measures rapidly shifted from flexible to rigid in the second half of 2020. In July 2020, the regulators adopted flexible measures against Internet platform companies and organized 20 Internet platforms in China to sign a letter of commitment. When the flexible regulatory measures did not work, the regulators carried out law enforcement investigations to reshape the order of market competition through tough regulatory measures. In December 2020, the State Administration for Market Regulation investigated Alibaba’s “either-or choice” policy.
- Fifth, there is timely response to market concerns and adjustment of regulatory style. Since the COVID-19 pandemic, China’s economy has faced difficulties and challenges, regulatory policies have been introduced intensively, mostly aimed at industries in which private enterprises are concentrated, and the “one size fits all” regulation has impacted on economic and social development; some foreign media have also taken this opportunity to speculate on the “crackdown on private enterprises.” The central government has responded and adjusted in a timely manner. The Central Economic Work Conference in December 2021 stated that the long-term goals should not be short-term and that the system goals should not be fragmented. Since 2022, the Chinese government has repeatedly expressed support for the balanced development of the platform economy and promotion of the legal compliance of the digital economy for domestic and overseas listing and financing, responding to domestic and foreign concerns regarding the suppression of the platform economy.^6^ The Wall Street Journal reported that “common prosperity,” a signature economic policy, aimed at reducing inequality, rattled businesses last year but has faded as China refocuses on shoring up growth.^7^

### Factors influencing regulatory policy practices in China

We list main factors affecting regulatory policy practices in China below:

- The first is the political system. The CPC and the Chinese government cater to the fundamental interests of the nation and the people, adhere to the people-centered philosophy of development, do not hesitate or waver in the face of short-term local interests (such as individual enterprises and investors), and resolutely take the necessary market regulation measures to achieve positive regulatory results. At the Sixth Plenary Session of the 19th CPC Central Committee, the Resolution of the CPC Central Committee on the Major Achievements and Historical Experience of the Party’s Centennial Struggle was adopted, stating that “the Party’s greatest political strength is its close ties with the people, while the biggest potential danger it faces as a governing party is becoming distanced from them” and that “development is for the people, depends on the people, and its fruits should be shared by the people. With unswerving resolve, we will pursue common prosperity for all”.
  Lin (2021) summarizes the internal structure of socialism with Chinese characteristics as a “dual and three-tier structure”: “dual” refers to socialism and the market economy, and “three tier” refers to a socio-economic structure in which the CPC is in a leading position, capital is in the middle, and labor plays a fundamental role; currently, the positive side of socialism should be better utilized, and the negative side of capital should be curbed.
  Under the people-centered philosophy of development, the socialist market economy takes into account the interests of multiple parties, does not maximize the interests of investors as the single goal, and integrates the needs of investors, employees, suppliers, consumers, communities, and national security in the development process. In contrast, there is an irreconcilable contradiction between Western neoliberalism and inclusive development, making it difficult to carry out major regulatory measures decisively.
- The second is leadership style. Chinese leaders are committed to a people-centered mindset and have a firm will to do so. According to a article in The Economist in August 2021, many US and European leaders are looking forward to shaping their technology industries according to President Xi’s vision: less social media and other “spiritual opium” and more strategic development of twenty-first century technology infrastructure, and they expect that if implemented properly, measures taken by the Chinese leaders will reduce inequality and set a good example for regulating large technology companies.
- The third is economic development stage. As an upper middle-income country, China should be and is in a position to implement high-quality development strategies and take proactive regulatory measures to build a “stakeholder” market economy model that is in line with the United Nations (UN) 2030 Agenda for Sustainable Development. Liu and Zang (2021) maintain that strengthening and improving market regulation is a critical requirement for the transformation of government functions in the new era, an important task for promoting the improvement of the national governance system and capacity and an important guarantee for accelerating the formation of a new development dynamic and promoting high-quality development.

### Aspects of China’s regulatory practice that need improvement

#### More importance attached to government regulation, but less to self-regulation of the industry

Government regulation and self-regulation of the industry are complementary and mutually reinforcing. Recent Chinese regulatory practices have mainly relied on the government’s “strong regulation” policy but lacks self-regulation of the industry, which actually increases the cost and reduces the efficiency of regulation, and does not give full play to the important role and enthusiasm of social forces, including enterprises.

#### More importance attached to rigid regulation, but less to flexible regulation

Huang et al. (2022) argues that improving the governance system is the basic prerequisite for the orderly development of the platform economy. It is necessary to correct the inappropriate behavior of the platform economy; additionally, it is necessary to complete the regulatory policy puzzle and establish a comprehensive governance system for the platform economy.

#### More importance attached to negative examples, but less to positive examples

It is necessary to establish models of self-adjustment, explain the conscious actions of enterprises representing different ownerships and different industries, and respond to rumors about crackdowns on private enterprises. Market regulation should be strengthened both strictly and with kindness. Enterprises, especially private enterprises, that take the initiative to strengthen self-regulation should be encouraged and supported, and their new practices and new philosophy should be actively promoted to the public.

#### More importance attached to administrative intervention, but less to the rule of law

It is necessary to continuously improve the economic governance system, focusing on the rule of law and multi-participation. Huang et al. argue that the simultaneous action of multiple departments likely results in “campaign-style” governance, which can cause a great blow to the platform economy. According to the latest data from the China Academy of Information and Communications Technology, Internet investment and financing in the first quarter of 2022 decreased by 76.7% year-on-year. The Wall Street Journal (Kubota 2022) reported that Chinese tech giants, including Alibaba and Tencent, have made massive layoffs in response to regulatory pressure from the Chinese government.^8^ Both practicing the new development philosophy and building a new market system require the active participation of market players. Currently, regulation is mainly based on administrative measures, and the next step should focus on promoting rule-of-law procedures. It is necessary to strengthen communication, increase policy transparency and predictability, and gain the trust of market players, especially domestic enterprises.

## Case analysis

China’s “regulatory storm” is in line with the new global exploration of improving the market economy system, especially the international framework of McKinsey’s “stakeholder” market economy (see Table 2). In this article, we illustrate China’s “regulatory storm” in terms of corporate governance, environmental goals, social impacts, adverse consequences, and employee rights, not only reflecting the people-centered paradigm of the state-market relationship but also is in line with the global reflection of the shortcomings of the current market economy system.

**Table 2:** China’s regulatory measures and the McKinsey framework of stakeholder principles

Basic elements	Corporate governance	Environmental goals	Social impacts	Adverse consequences	Employee rights
**Connotations of McKinsey’s framework**	Focus on the interests of multiple parties, add corresponding independent directors, and prioritize the interests of stakeholders rather than investors	Formulate and implement environmental protection goals	Enhance suppliers’ capacity to promote their care for economic, environmental, and social impacts; promote inclusive development and strengthen interaction with communities	Serve the long-term needs of consumers and eliminate the foreseeable negative consequences of products	Respect employees’ rights and invest in their future
**China’s regulatory measures**	Reform of private enterprise governance and strengthening of corporate social responsibility	“Coal exit” measures for financial institutions and enterprises	Antitrust and inclusive development measures	Measures to strengthen the protection of minors	Protection measures for vulnerable employees

Source: Authors

### Corporate governance

China’s regulatory practice emphasizes the integration of CPC organization into the corporate governance structure and the incorporation of corporate social responsibility into the corporate development strategy and governance structure, which is consistent with the philosophy of valuing multi-interests in the international framework.

#### Integration of CPC organization into corporate governance structure

Advocated by the central government, private enterprises set up grassroots Party organizations to promote enterprises and employees into a community with a shared future, assume more social responsibilities, and facilitate the balanced and sustainable development of the private economy. Since the 18th National Congress of the CPC, the CPC Central Committee has put forward a series of major ideas and measures to strengthen the Party initiatives for private enterprises. Under the leadership of Party committees at all levels and the guidance of organization departments, private enterprises have continuously promoted “two coverages,” i.e., organizational coverage and work coverage. Thus far, the Party’s organization and work have achieved full coverage of key enterprises and key regions. The development practices of many enterprises have proven that, whether in daily operation or when facing a crisis, insisting on arming employees with the Party’s ideology can guide cadres and workers to improve their personal qualities, establish higher values, cultivate the spirit and creed internalized by employees, shape a common vision with enterprises, and make enterprises and employees become a community of shared future. Within the enterprise, under the Party guidance, integrity and self-discipline are strengthened to ensure the healthy development of private enterprises, so as to lay a solid foundation for the development of the private economy. Externally, Party-building work has also promoted enterprises to establish a strong sense of responsibility and assume more social responsibilities.

#### Incorporating corporate social responsibility into corporate development strategy and governance structure

Under the active advocacy of the Chinese government, well-known private enterprises, such as the Yili Group, have set up “sustainability committees” to align with sustainable development goals, unite upstream and downstream partners, and take into account the interests of multiple parties, with a similar effect to that of corporate governance reform suggested by McKinsey. The Yili Group has benchmarked the sustainable development goals and selected nine goals for inclusion in the group’s development strategy. It has also established a three-tier system, including a group-level sustainable development committee, a secretariat under the committee, and sustainable development liaison officers in every department and business line. Additionally, the Yili Group works together with partners upstream and downstream in the industry chain, including the planting and breeding industries, the logistics and processing industries, and the consumer service industry, spanning primary, secondary and tertiary industries, to excel in sustainable development. Corporate social responsibility is a system that starts from the strategic level and permeates daily business operations and is integrated with corporate development, industry development, and ecological chain development.

### Environmental goals

#### China’s regulatory practices emphasize the “coal exit” measures of Chinese financial institutions and enterprises, consistent with the philosophy of environmental protection and sustainable development in the international framework

In September 2021, Chinese leaders officially announced that they would no longer build new overseas coal-fired power projects. In fact, prior to that, the Chinese government and enterprises had already adopted strict environmental standards in their overseas investment and financing projects. At the ministerial level, in 2017, the National Development and Reform Commission passed the Administrative Measures for the Outbound Investment by Enterprises, which requires “focusing on ecological and environmental protection and establishment of a good image of Chinese investors”. In 2017, the Ministry of Ecology and Environment adopted the Guidance on Promoting Green Belt and Road, which requires “paying attention to the ecological and environmental protection demands of local people.” At the level of financial institutions, in 2012, the China Banking Regulatory Commission passed the Green Credit Guidelines, which requires banks to pay attention to environmental protection and land laws/regulations in the countries where the projects are located; Article 21 specifically requires banks to adopt relevant UN international practices or international guidelines and best practices. Currently, major domestic financing institutions, including policy banks such as the Export-Import Bank of China and commercial banks such as the Industrial and Commercial Bank of China (ICBC) have adopted the green “one-vote veto system” for overseas loan projects. In recent years, the ICBC has withdrawn twice from coal power financing programs in Africa, amounting to $5 billion. At the level of social institutions and industry associations, in 2018, the Green Finance Committee of the China Finance Society adopted the “Belt and Road” Green Investment Principles, setting out seven principles.^9^

### Social impacts

#### China’s regulatory practice emphasizes antitrust measures, which fits in with the international framework’s philosophy of antitrust and inclusive development

The main area of antitrust is to strengthen the regulation of the disorderly expansion of Internet platform enterprises and prevent winner-take-all behavior such as the crowding out of small and medium-sized enterprises by leading platform enterprises in the industry and the crowding out of small traders cross industries. The Central Economic Work Conference held in December 2020 specified eight key tasks in the economic work in 2021, and “strengthening antitrust and preventing the disorderly expansion of capital” was listed as one of them, which was further emphasized at the Central Economic Work Conference in December 2021. Since the end of 2020, a number of documents have been issued at the national level to guide and standardize the development of the platform economy, including both encouraging documents such as the Guiding Opinions on Promoting the Standardized and Healthy Development of the Platform Economy issued by the General Office of the State Council, and binding documents from various ministries aimed at addressing specific problems, such as the Guiding Opinions on Strengthening the Comprehensive Governance of Network Information Service Algorithms and the Opinions on Further Regulating the Profit-making Behavior of Webcasting to Promote the Healthy Development of the Industry jointly issued by the Central Cyberspace Administration of China and other ministries. In addition, there are some guidance documents that have been released or are under development, such as the Guidelines for Antitrust in the Field of Platform Economy by the Antitrust Committee of the State Council and the Guidelines for the Classification and Grading of Internet Platforms and the Guidelines for the Implementation of the Entities’ Responsibility of Internet Platforms by the State Administration for Market Regulation. These documents have basically built a policy system for the regulation of the platform economy in China and established the policy orientation of attaching equal importance to the development and regulation of the platform economy. Since 2022, relevant regulatory measures have continued to intensify. The Central Cyberspace Administration of China has taken the lead in carrying out a campaign regarding regulation of platform economy. The aim is that, by early December 2022, it will thoroughly investigate and rectify the security problem of platform algorithms of Internet companies, focusing on large websites, platforms, and products with strong public opinion attributes or social mobilization capabilities and urging companies to use algorithms to increase the dissemination of positive energy, dispose of illegal and undesirable information, and rectify the chaos of algorithm abuse.

### Adverse consequences

#### In terms of adverse consequences, China’s regulatory practices emphasize measures to strengthen the protection of children, consistent with the philosophy of eliminating foreseeable adverse consequences in the international framework

Market regulation is exemplified by the recent rectification of the educational training industry to significantly reduce the extracurricular burden on students and the recent rectification of “fan bases” to strictly control the luring of minors to participate in related consumption and undesirable activities. In July 2021, the General Office of the CPC Central Committee and the General Office of the State Council issued the Opinions on Further Reducing the Burden of Homework and After-school Tutoring on Students in Compulsory Education and required that all regions and departments implement it according to the actual situation. This document has had a considerable impact on the educational training industry and clearly stipulates the access qualifications, financing means, business hours and marketing methods of online and offline educational training institutions. For example, starting in November 2021, New Oriental no longer provided subject-related training services to students from kindergarten to ninth grade. In addition, in 2021, the Central Cyberspace Administration of China deployed a series of 15 special tasks in “Clearance Operation,” including the rectification of chaotic “fan bases,” with a total of more than 22 million pieces of illegal and undesirable information cleaned up, 1.34 billion accounts disposed of, over 7200 livestream anchors banned, over 2160 apps and applets removed, and over 3200 websites closed.^10^

In terms of market self-regulation, for example, Tencent independently strengthened the protection of minors in games. In the game industry, the phenomenon of developing software targeting human weakness has already crossed enterprises’ ethical bottom line. Leading companies such as Tencent have reflected on this matter seriously and taken practical measures to strengthen the protection of minors in their games.

### Employee rights

#### In terms of employee rights, China’s regulatory practices emphasize protection measures for vulnerable employees, consistent with the philosophy of safeguarding employees’ vital interests in the international framework

For example, seven ministries, including the State Administration for Market Regulation, jointly required the protection of the legitimate rights of food delivery workers. In July 2021, the seven ministries jointly required that the “strictest algorithm” not be used as an assessment requirement, that assessment factors such as the number of orders, punctuality rate, and online rate be reasonably determined through methods such as an “algorithm that considers both platform efficiency and worker protection,” and that the delivery time limit be appropriately relaxed. Eight ministries, including the Ministry of Human Resources and Social Security, jointly issued the Guiding Opinions on Safeguarding the Rights of Workers in New Employment Forms, which clarifies that platform enterprises should take responsibility for the protection of the rights of workers in new forms of employment. The introduction of the above opinions also put forward a variety of requirements for the online catering platform, which can be described as a tailor-made “armor” for the protection of rights of food delivery workers.

## Applicability of China’s regulatory experience: implications for other Asian countries

China’s recent regulatory measures reflect the effectiveness of the “strong government” in rapidly rectifying market order and are in line with the global trend of building a new market system represented by the “stakeholder” market economy, which is highly compatible with the 2030 Agenda for Sustainable Development and worthy of reference for other Asian countries.

### Other Asian countries are similar to China in terms of political and economic development paradigms and have a tradition of “strong government”

The common development experience of Asian countries mainly includes meritocracy, people-centeredness, collective values, the philosophy of development first, and the unification of an effective government and efficient market. The development of China and other Asian countries has in fact overturned the World Bank’s assertion that the “East Asian miracle” is just a replica of the Washington Consensus. China’s “strong government” and “strong regulation” have certain reference significance for other Asian countries, especially for East Asian countries with a strong government, in dealing with state-market relations.

### Asian countries could learn from but not copy China’s regulatory experience

Asian countries have their own unique political and economic systems, and when drawing on global and Chinese experiences, it is advisable to formulate their own regulatory priorities, tools, and transition timetables that are appropriate for them, rather than directly copying other’s experiences. In particular, in view of the shortcomings in China’s regulatory policy framework, such as imperfect regulatory governance, insufficient establishment of positive examples, and insufficient rule-of-law construction, they should plan ahead to avoid the economic and social risks brought about by multipronged and campaign-style approaches.

### China and other Asian countries should strengthen exchanges and cooperation in economic governance reform

China and other Asian countries share common values such as sustainable development and inclusive development and should strengthen policy communication and experience exchange, expand the convergence of interests, and cooperate to address challenges. In particular, China and other Asian countries can exchange and learn from each other on how to improve the state-market relationship and how to develop regulatory strategies that are in line with their own political systems and economic and social development stages.

## Data Availability

Not applicable.

## Footnotes

1 http://opinion.people.com.cn/n1/2021/0908/c1003-32220675.html.

2 https://researchprofiles.canberra.edu.au/en/publications/smart-regulation.

3 https://www.bjdj.gov.cn/article/8395.html.

4 http://www.nopss.gov.cn/n1/2021/1011/c219544-32249570.html

5 https://cn.wsj.com/articles/%E8%85%BE%E8%AE%AF2021%E5%B9%B4%E7%AC%AC%E5%9B%9B%E5%AD%A3%E5%BA%A6%E5%88%9B%E8%BF%91%E4%BA%8C%E5%8D%81%E5%B9%B4%E6%9D%A5%E6%9C%80%E4%BD%8E%E6%94%B6%E5%85%A5%E5%A2%9E%E9%80%9F-11648032665?mod=ig_chinatechregulatorycn.

6 In May 2022, Liu He stated at a consultation meeting on the topic of “promoting the sustainable and healthy development of the digital economy” that it is necessary to support the sustainable and healthy development of the platform economy and the private economy and study specific measures to support the standardized and healthy development of the platform economy and that it is necessary to well handle the relationship between the government and the market, coordinate planning development, improve laws and regulations, and support the listing of digital enterprises in domestic and international capital markets.

7 https://www.wsj.com/articles/xi-jinpings-common-prosperity-was-everywhere-but-china-backed-off-11648978380

8 https://cn.wsj.com/articles/%E4%B8%AD%E5%9B%BD%E7%A7%91%E6%8A%80%E5%B7%A8%E5%A4%B4%E5%A4%A7%E5%B9%85%E8%A3%81%E5%91%98%E5%BA%94%E5%AF%B9%E7%BB%8F%E6%B5%8E%E6%94%BE%E7%BC%93%E5%92%8C%E7%9B%91%E7%AE%A1%E5%8E%8B%E5%8A%9B-11647917461?mod=ig_chinatechregulatorycn

9 Namely, incorporating sustainability into corporate governance, fully understanding ESG risks, fully disclosing environmental information, strengthening communication with stakeholders, fully using green financial tools, adopting green supply chain management, and building capacity through multiparty cooperation.

10 http://www.cac.gov.cn/2022-03/17/c_1649125522577850.htm
